# Case of Aneurism

**Published:** 1808-09

**Authors:** Joshua E. White

**Affiliations:** Waynesborough, in America


### CASE *of* ANEURISM; *by Dr.* JOSHUA E. WHITE, *of Waynesborough, in America.*

ON Thursday, the 23d of May, 1799, I was requested to visit Mrs. P. aged 55 years, living a few miles from this place, who, since Christmas last, had been troubled with a tumour under the upper part of the sternum, which her friends supposed to be what is commonly called a wen, and for the extirpation of which I was called. During the last three weeks the swelling had increased rapidly in size. The integuments were much distended; the cutis had changed colour, and become of a very deep red. Upon examination I found it to be immoveable, of a firm consistence, and the surface smooth and equal. Throughout the whole of it a strong and violent pulsation was to be felt, which was evident to the sight at the distance of several feet. Its nature, unfortunately for the patient, was too evident, and I did not hesitate to pronounce it to be an aneurism. The patient suffered much pain, not only from the tension of the skin, but from its weight. She could not lie down with any tolerable ease, and she had shooting pains in different parts of her breast. The tumour was about ten inches in circumference round the base, and the contents could not be made to recede the least upon pressure. A few days previous to my seeing her, two small punctures had been made in the anterior part of the swelling, ing, at her request, upon the supposition of its being an abscess, and with a view of discharging its contents. Neither of them penetrated the aneurismal sac, though one of them divided the external coat of the artery; for at every diastole of the heart, the thin internal coat (which might here be emphatically called the "thread of life") was evidently pushed forward, and seemed every moment ready to yield to the *vis à tergo*. The discharge of blood from the vessels of the skin afforded her considerable relief from pain and tension.

The patient could not trace the origin of the tumour to any certain cause: hence it most probably arose from a partial weakness of the aorta. She had been for many years affected with the chronic rheumatism. About twelve months before the present tumour made its appearance, a smaller one had discovered itself in the same place; but which, in a short time, disappeared, without any application. With the complicated distress of pain, restless nights, and unpleasant forebodings, the patient was anxious for relief, and entreated me to do something towards it. For the removal of the tumour, no remedies, I told her, could be applied; and her friends were made acquainted with its nature, and the inevitable result. To support it against the violent impulse of the blood, I advised a covering of thin sheet lead: rest was strongly enjoined, and anodynes were occasionally directed. Expecting the tumour would soon burst, and prove instantly fatal, I left her. This event took place in a week. The discharge of blood was so enormous and sudden, that the by-standers compared it to its being "poured from a pail suddenly turned upside down." The orifice was about the size of a dollar. She expired without a groan, and in an instant: permission could not be gained to open the body. Thus we are left in ignorance as to the effects produced upon the lungs, &c. There was an evident separation of the clavicles from the sternum (most probably from an absorption of their substance), and this last was considerably elevated. The hardness which I have noticed most probably arose from a deposition of matter which sometimes takes place round an aneurismal sac, and which, in some instances, has been known to approximate to a cartilaginous or osseous nature. The person who made the punctures said, "it cut like gristle."

That the action of an artery should be able to produce such a derangement or loss of substance as appeared to be evident in this case, not only in the clavicles, but also in the sternum and ends of the ribs, may, at first view, appear surprising; but our wonder will cease when we know that the most violent effects are sometimes produced upon bones, as well as the softer parts, by the pulsation of a large artery in an aneurism; that the former may be, and often are, separated from their joints, elevated or displaced from their natural situation, and sometimes entirely dissolved.

